# Early signs of tinnitus in a simulation of the mammalian primary auditory cortex

**DOI:** 10.1186/1471-2202-12-S1-P383

**Published:** 2011-07-18

**Authors:** Christoph Metzner, Melea Menzinger, Achim Schweikard, Bartosz Zurowski

**Affiliations:** 1 Institute for Robotics and Cognitive Systems, University of Luebeck, 23538 Luebeck, Germany; 2 Graduate School for Computing in Medicine and Life Sciences, University of Luebeck, 23538 Luebeck, Germany; 3 Department of Psychiatry, University Clinics Schleswig-Holstein, 23538 Luebeck, Germany

## 

The majority of tinnitus cases are related to cochlear dysfunction, leading to altered peripheral input to the central auditory system [1]. These alterations are believed to increase the basic level of neural activity during off-conditions of sound and to diminish the increase in neural activity when sound is presented [2]. As a compensatory means the affected region of primary auditory cortex tries to maximize the difference between basic level activity and sound-induced activity by adapting inhibitory and excitatory influences towards less GABAergic inhibition. This adaptation in turn triggers unmasking of dormant synapses and creation of new connections through axonal sprouting and finally results in a reorganization of tonotopic receptive fields and the manifestation of tinnitus [3].

To further investigate the processes involved in tinnitus manifestation we used neuroConstruct [4] to implement a simulation of the primary auditory cortex. It consists of two groups of different types of neurons, excitatory regular spiking pyramidal cells and inhibitory fast spiking basket cells with a group size of ? and ?, respectively. Its organization is based on experimental data from animal studies. Neurons were modeled as conductance-based multi-compartment neurons having numerous ionic conductances to achieve the desired firing properties. Model neurons posses glutamate- (AMPA and NMDA) and GABA-sensitive synaptic receptors.

Afferent input from the auditory pathway was modeled as trains of spikes. Their frequency depended on the strength of the input whereas their target site depended on the frequency of the input. Additionally, synaptic background noise was introduced to the system as Gaussian noise. Excitatory cells made connections to other excitatory in their 1st, 2nd and 3rd surrounding ring and to inhibitory cells in their 3rd, 4th and 5th surrounding ring with decreasing probabilities (1.0, 0.8, 0.6 respectively). Inhibitory cells made connections with excitatory cells in their 1st, 2nd, 3rd and 4th surrounding ring allso with decreasing probability (1.0, 0.8, 0.6, 0.5 respectively). This connectivity pattern results in a tonotopic organization of the auditory cortex layer. The simulation also exhibits spontaneous and spike driven firing rates comparable with experimental data [5].

Increasing the background noise and simultaneously decreasing the afferent input to one tonotopic region creates a state that may correspond to the early stage of tinnitus manifestation after cochlear damage. This state also shows an increased basic level of activity in the absence of input and furthermore, a diminished increase of activity during sound presentation. Moreover, a change of the ratio between excitatory and inhibitory weights towards more excitation and less inhibition results in a normal increase of activity during sound presentation. This suggests that the auditory cortex might react upon this diminished ability to detect sound in the damaged frequency region by increasing excitation and decreasing inhibition.

The presented simulation constitutes an excellent starting point for a deeper investigation of the early phases in the generation of tinnitus. We are currently extending the simulation to include more structures of the classical pathway and to incorporate synaptic plasticity.
